# Litho-structural interpretation of aeromagnetic anomalies reveals potential for mineral exploration in Tizi n'Test Region, Western High Atlas, Morocco

**DOI:** 10.1038/s41598-024-65941-1

**Published:** 2024-07-24

**Authors:** Bouchra Dadi, Farid Faik, Said Boutaleb, El Hassan Abia, Driss El Azzab, Mohammed Ouchchen, Younes Mamouch, Fatima Zahra Echogdali, Kevin L. Mickus, Mohamed Abioui, Mohamed Sobh, Soha Hassan

**Affiliations:** 1 Department of Earth Sciences, Faculty of Sciences, Ibn Zohr University, Agadir, Morocco; 2 InterDisciplinary Applied Research Laboratory - LIDRA, International University of Agadir - Universiapolis, Agadir, Morocco; 3 Laboratory of Intelligent Systems Geo-Resources and Renewable Energies, Faculty of Sciences and Technology, Sidi Mohamed Ben Abdellah University, Fez, Morocco; 4 PCPM Laboratory, Research Team of Geology of the Mining and Energetics Resources, Faculty of Sciences and Technology, Hassan First University of Settat, Settat, Morocco; 5 Department of Geosciences, Missouri State University, Springfield, MO USA; 6 Department of Earth Sciences, Faculty of Sciences and Technology, MARE-Marine and Environmental Sciences Centre - Sedimentary Geology Group, University of Coimbra, Coimbra, Portugal; 7 Leibniz Institute for Applied Geophysics (LIAG), Hannover, Germany; 8 Department of Geodynamics, National Research Institute of Astronomy and Geophysics (NRIAG), Helwan, Egypt

**Keywords:** Aeromagnetic data, Structural analysis, Mineral exploration, Tizi n'Test, Western High Atlas, Morocco, Geology, Geophysics, Mineralogy

## Abstract

This study interprets aeromagnetic data from the Tizi n'Test area in the High Atlas massif of Morocco, aiming to gain insights into its litho-structural architecture and implications for mineral exploration and mining. We employed six different analytical techniques to the residual magnetic field data, including reduction to the pole (RTP), upward continuation, total horizontal derivative, Tilt angle, Centre for Exploration Targeting (CET) analysis, and Euler deconvolution. Our analyses differentiated the study area into three magnetic domains: the eastern Ouzellarh block, characterized by positive anomalies, a central domain characterized by a negative magnetic signature demarcating the transitional zone between the Anti-Atlas and the High Atlas separated by the Ouchden fault: and the western domain, represented by the Tichka massif. The application of total horizontal derivative, tilt angle, and a combination of filters in ternary image formats (Tilt angle, upward continuation 1000 + Tilt angle and upward continuation 3000 + Tilt angle) revealed both known and previously unidentified geological lineaments, mapping structural complexity across various orientations (NE–SW, NNE–SSE, E–W, NW–SE, and N–S). The CET grid analysis method unveiled the structural complexity, highlighting the geodynamic evolution of the region. Particularly, the Ouchden fault delineates a magnetic domain divide between the ancient High Atlas and the Ouzellarh block (Anti-Atlas). Furthermore, Euler deconvolution indicated magnetic source depths ranging from 52 m in the western domain of the Tichka massif to 6560 m in the Ouzellarh block. A comprehensive structural scheme, classified by C-A fractal analysis, identified zones favourable for exploration and mining, particularly along the Ouchden fault, Tizi n'Test, NE-SW trending lineaments in the northwestern domain, as well as along the Tichka granite’s margin.

## Introduction

The Tizi n'Test area is pivotal for analysing the structural architecture of the Moroccan Hercynides due to its position as a hinge between the Anti-Atlas and Meso-Atlas domains, which diverged structurally during the Variscan orogeny. Despite hosting several ore deposits and prospects, such as the Upper Seksaoua copper and barite deposit^1^, Taghouacht barite and albitite deposit^2^, and Tichka granitoid-related peribatholitic Mo and Au mineralization^3^ (see Section "Mineralization"), the area’s challenging accessibility has limited through exploration efforts. From this perspective, analysing processed airborne magnetic data available over the area can be useful in delineating additional ore deposits^4^.

Enhancing geophysical data through various magnetic mapping methods has significantly improved the structural interpretation, enabling the detailed mapping of faults, folds, shear zones, and magmatic intrusions^5^. Enhancement methods such as the vertical derivative^6^, analytic signal amplitude^7^, total horizontal derivative^8^, horizontal Tilt angle^9^, Tilt angle^10^, Theta Map^11^, Centre for Exploration Targeting grid analysis technique^12^, and Euler deconvolution^13^ have been used. These enhancement methods not only facilitate the mapping of magnetic sources and their depth estimation but also help in understanding the study area structural framework and identifying potential mining zone^14,15^.

The generating of magnetic lineament maps, indicative of structural features, intrusions or hydrothermal events, plays a crucial role in delineating brittle tectonic structures, potentially acting as conduits during magmatic or hydrothermal processes^16^. These structures are critical in understanding the spatial distribution of ore deposits, including base, precious, and rare earth metals^17^, which are often elucidated through fractal/multifractal modelling^18^.

This paper focuses on six different methods (e.g., filters) to enhance magnetic data: (i) reduction to the north pole (RTP) of the total magnetic field, (ii) upward continuation, (iii) total horizontal derivative (iv) tilt angle, (v) Centre for Exploration Targeting grid analysis, and (vi) Euler deconvolution. With the support of these enhancement methods (e.g. filter), we present a structural interpretation of the area, establishing spatial correlations among outcrops, magnetic lineaments, and potential ore deposits, employing a concentration area (CA) modelling. This study's outcomes significantly contribute to identifying regions with heightened ore deposition potential, offering valuable insights for formulating exploration programs.

## Geological background

### Geological and structural framework

**Figure 1:**
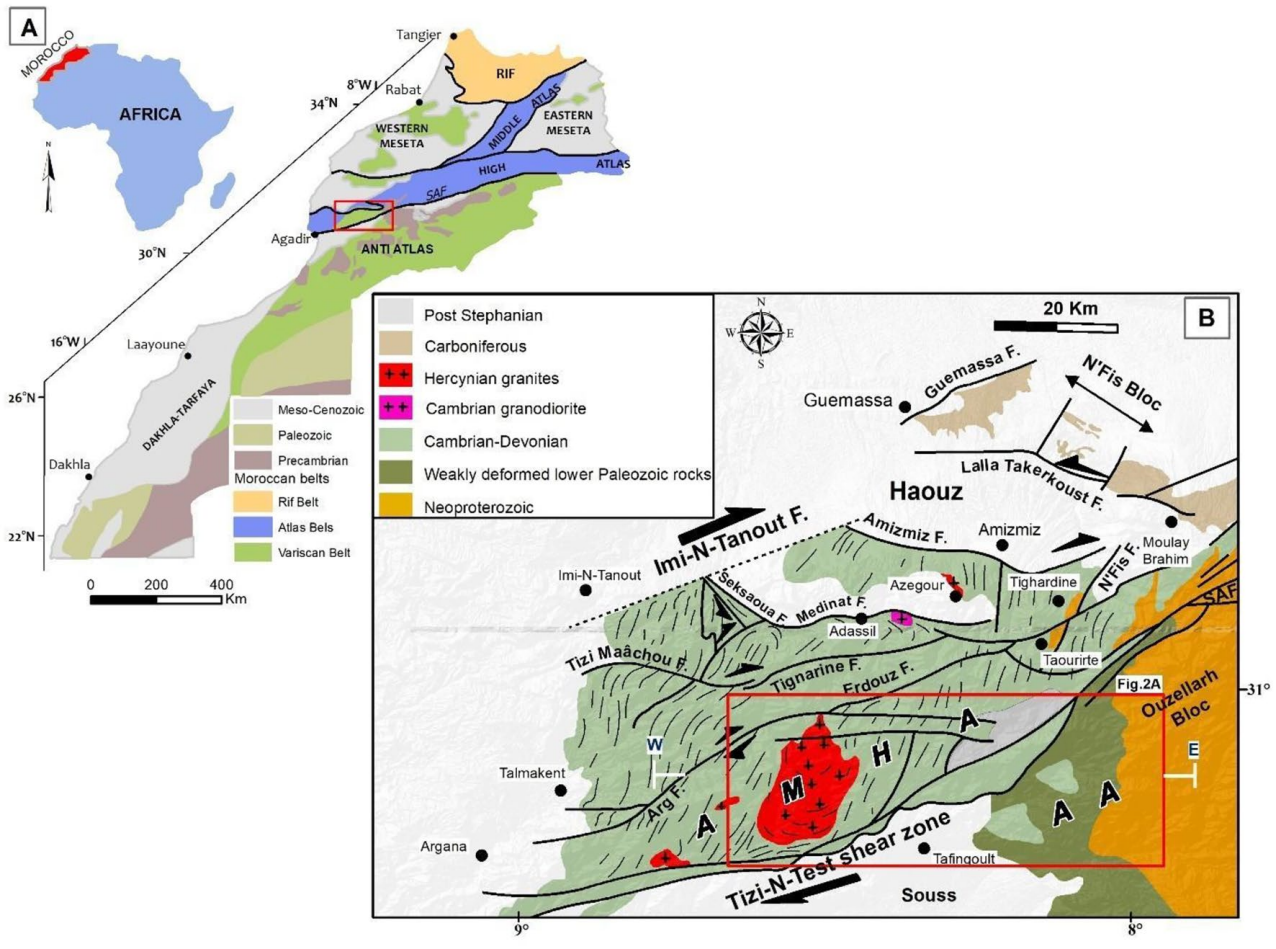
Overview of Geographical and Geological Context. (**A**) Morocco's structural domains with the highlighted location of the Tizi n'Test region within. (**B**) Simplified geological map of the Western High Atlas and location of the study areas, based on the works of^46^. Key features include the Ancient Massif High Atlas (AMHA), Anti Atlas (AA), and South Atlasic Fault (SAF). The map was created using ArcGIS, version 10.6.1, (https://www.esri.com/en-us/arcgis/about-arcgis/overview).

**Figure 2:**
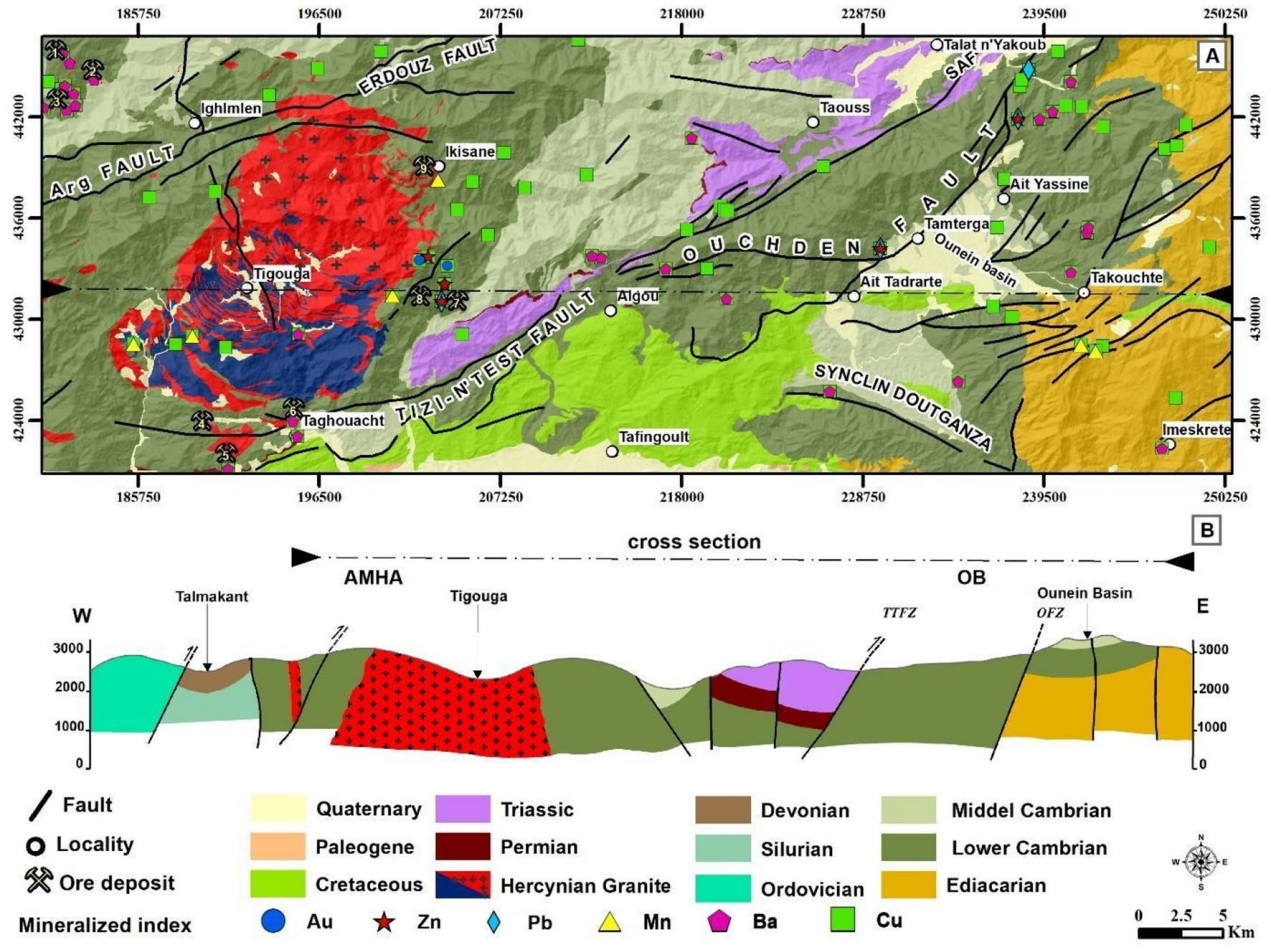
Geological map and Cross-Section of the Tizi n'Test Region (**A**) Detailed geological map of the study area highlighting key localities: 1—Tirquine, 2—Talat n’Tiouia, 3—Ifri n’Janjar, 4—Goudacha, 5—Jbel Boufounas, 6—Taghwacht, 7—Jeralna, 8—Alebdi, 9—Ikisane. (**B**) Illustrates a geological cross-section of the study area, showing a major geological blocks and fault zones including the Ouezllarh Block (OB); Ancient Massif High Atlas (AMHA), Ouchden Fault Zone (OFZ), Tizi n’Test Fault Zone (TTFZ). The maps were generated using ArcGIS, version 10.6.1.

The Tizi n'Test region is located in the western Paleozoic block of the ancient High Atlas massif (AMHA), aligns westward with the Meseta^19^ and intersects the Ouzellarh Block (OB). This latter feature juts into the Meso-Atlas domain as a promontory from the Anti-Atlas in (Fig. 1A,B)^20^ delineated by the South Atlas Fault which demarcates the Anti-Atlas is to the south from the High Atlas to the north^21^. The study area includes the southern part of the Ouzellarh block, marked by ancient volcanic and sedimentary rocks crossed by Hercynian batholiths along the Tizi n'Test fault, stretching southeast, and the Erdouz-Arg fault in the northwest. The western part of the AMHA block has a covering of volcanic and sedimentary rocks from the upper Ediacaran Paleozoic period, with a major unconformity above the Precambrian basement that extends towards the SE to constitute the Sirwa massif. The Ediacaran terrains include rhyolitic and andesitic lavas and tuffs invaded by granites, gabbros^22^ and later by felsic, intermediate, and basic dykes^23^. In the Ounein Basin (Fig. 2B) of the Ouzellarh block, the Cambrian formations exhibit a slight unconformity or conformity with the Ediacaran. These Cambrian formations, exposed in various regions, are separated by ENE-WSW faults (Fig. 2A). Lower Cambrian units comprise dolomites with stromatolites, interbedded with basaltic flows, transitioning to olive-colored shales with Paradoxides fauna, underlain by brecciated limestones analogously associated with the Micmacca breccias of the Anti-Atlas, attributed to the Middle Cambrian^24^.

Moussu in 1959^25^ estimated that the Cambrian units in this area are less than 1 km thick, increasing towards the southeast in the Adrar Doutganza syncline in the Anti-Atlas (Fig. 2A). In the western AMHA block, between the Argana Corridor and the Ouchden Fault, the Paleozoic rock layers range from the Cambrian to the Devonian (Fig. 2A). In the western block of the AMHA, between the Argana Corridor and the Ouchden Fault, the Paleozoic stratigraphy extends from the Cambrian to Devonian (Fig. 2A)^26^. Similarities are observed between Cambrian formations in this region and those found in the eastern part of the Ouzellarh block, where volcanic rocks and pyroclastic materials are prevalent^27^. Younger Paleozoic rocks (Ordovician to Devonian) are only present in the western part, within small-scale synclines in the Talmakant area (Fig. 2B). Ordovician deposits consist of clayey-sandstone mixed with quartzite^26,28^, shales and phtanites represent Silurian^29^, and Devonian units include calcareous or sandstone turbidites^30^. The western block, heavily deformed due to involvement in multiple orogenic events, has an estimated thickness of about 5 km^31^.

**Figure 3:**
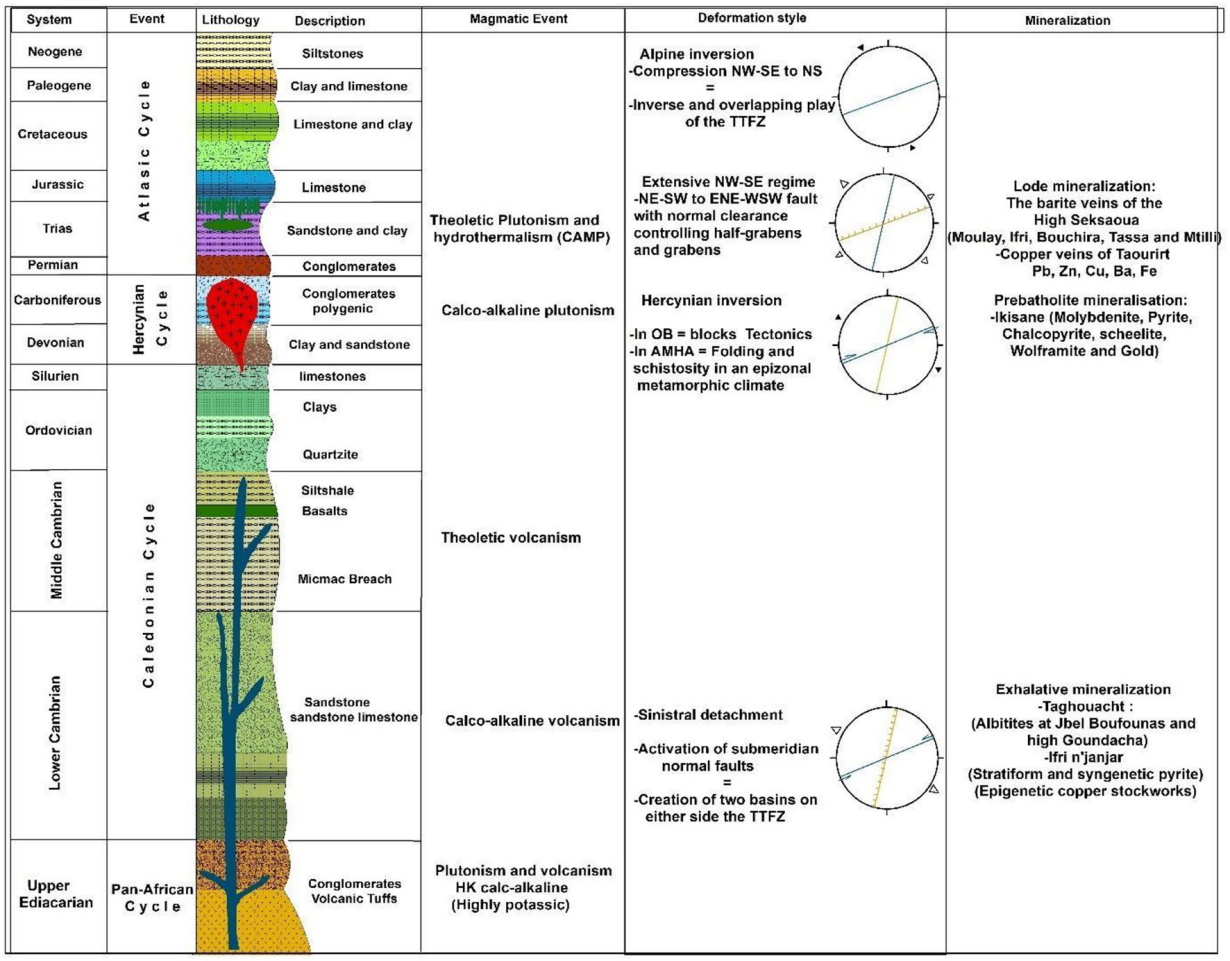
Lithostratigraphic column of the geological formations of the ancient massif of the Western High Atlas with the main tectono-magmatic events and associated ore deposit^26,28,38,39,44,50^.

At Jbel Tichka, Cambrian units are intruded by Hercynian-aged (291 ± 5 Ma) granitoid rocks^32^, accompanied by a series of dykes^33^. Mafic dykes undeformed mafic dykes are ubiquitously distributed across the study area, hypothesized to have been emplaced subsequent to the peak Hercynian orogeny (Fig. 3). Their likely association with the extension related to the Central Atlantic Magmatic Province (CAMP) during the Triassic is suggested^15^. The Triassic units within the study area comprise epicontinental red-coloured clastic, including siltstones, sandstones, and conglomerates, which are preserved within minor basins bounded by and associated with the South Atlas Fault^34^. In the Argana Corridor west of the study area, these deposits are interbedded with tholeiitic- basalts dated at 199 Ma (^40^Ar/^39^Ar)^35^. Cretaceous units outcroppings in the southern portion of the study area consist of siliciclastic deposits interbedded with carbonate unit bars^36^.

During the Variscan orogeny, the AMHA and OB blocks responded disparately to tectonic forces. The OB exhibited reactivation within rigid blocks, mirroring the observed style in the Anti-Atlas^37^. Deformation in the OB was constrained to the reactivation of fractures inherited from the basement during Lower Cambrian rifting, characterised by reverse faults observed in open folds without penetrative schistosity or metamorphism. Conversely, in the western AMHA block, deformation led to the development of schists under epizonal conditions, accompanied by NNE-SSW trending schistosity reminiscent of that observed in the Paleozoic terrains of the Western Meseta^26,38^.

The intrusion of the Tichka massif (Fig. 3) is associated with a halo of thermal metamorphism. Tectonic forces gave rise to two principal structural trends: an ENE-WSW trend parallel to the orientation of the Atlas Range and an NNE-SSW trend aligned with structures formed during the Variscan orogeny. The ENE-WSW trends, demarcating the Western Block and the Ouzallarh Block, constitute a significant original lineament^19^.

A thorough examination of the existing geological data^38,39^, reveals that the studied region constitutes a polyorogenic zone. In this intricate geological setting, structural trends are presumed to have been inherited from pre-existing structures within the Precambrian basement, undergoing multiple reactivations across the region's polyphase history. The primary reactivation events are as follows:

- During the Lower Cambrian, the lineament acted as a transfer fault^40^ separating two aborted intracontinental basins controlled by NNE-SSW trending normal faults; The Western High Atlas basin in the north^41^, and The Western Anti-Atlas basin in the south. The separation of these grabens is associated with the opening of the Iapetus Ocean, dividing the North American craton and the Gondwana-Avalon supercontinent^42^. This tectonic event also involves the outpouring of magnetic material and hydrothermal activity^2^.
- In the course of the Permian-aged Variscan orogeny, prominent lineament faults assumed a syn-schist dexterous structure^43^ exerting control over the emplacement of the Tichka pluton and the concomitant mineralization processes^44^.
- In the Triassic-Lias, the opening of the Atlantic ocean formed a series semi-grabens whose orientations were controlled by the lineaments^39,43^. Like the other regions of the CAMP (Fig. 3), the High Atlas corresponds to an aborted intracontinental rift and also contains basaltic flows (basalts of the Argana corridor) and doleritic dykes^39^.
- During the Toarcian epoch, persistent structural trends are observed^39,45^ likely linked to the separation of the African plate from the European plate. The extensions of these lineaments towards the central High Atlas fragment played a pivotal role in controlling the emplacement of Jurassic magmatic intrusions^45^.
- In the Neogene period, Alpine inversion processes reactivated the lineaments, transforming them into overlapping reverse faults^38^and transitioning into a transpressive regime^30^.

### Mineralization

The mineralization within the study area is categorized into three distinct groups based on metallogenic overviews and field observations:

*Exhalative mineralization* associated with Cambrian volcanism, is situated within an extensional setting during the formation of Lower Cambrian basins^46^. This tectonic–magmatic event is notably linked to a submarine hydrothermal occurrence at Taghouacht, along the southern periphery of the Tichka massif (Figs. 2 and 3), wherein a thirty-meter-thick barite deposit occurs^32^. Barite is found in the volcanic host rocks (rhyolites) and mineralized sandstone beds, accompanied by chalcopyrite, quartz, white mica, tourmaline, and rutile as accessory minerals. This mineralization is confined within the Lower Cambrian units^33^, and outcrops in a region that extends from east to west for more than 5 km^2^, showed that sodic alteration of the rhyolite could be related to a late hydrothermal episode during Cambrian volcanism. In this region and along the Tizi n'Test lineament, the Lower Cambrian volcanic and volcano-sedimentary formations underwent albitization through metasomatism^2^. Moreover, several albitite outcrops scattered along this lineament are now being exploited at Taghouacht, Jbel Boufounas, and Haute Goudacha. Further to the northwest, the Ifri n'Janjar deposit, hosted in Lower Cambrian volcano-sedimentary units, contains a stratiform and syngenetic pyritic ore and an epigenetic stockwork copper ore. Initially perceived as a Volcanogenic Massive Sulfide (VMS) type deposit^47,48^, further analysis revealed the presence of uranium (U), tungsten (W), and tin (Sn) in the epigenetic paragenesis, as well as a Pb/Pb dating of brannerite indicating an age range between 264 ± 67 and 300 ± 10 million years ago (Ma). This dating coincides with the period of copper mineralization. These findings strongly suggest the involvement of the Tichka granite in the genesis of the deposit^49^. In general, the Lower Cambrian is marked for its rich exhalative-type mineralization on a global scale. The mineralizing fluids were channelled by faults involved in the extension during the early formation of the Cambrian basins.

*Peribatholitic mineralization* associated with the Tichka granitoid, positioned toward the conclusion of the Variscan event (Fig. 3). Pneumatholites formed during the later stages of intrusion led to pyrometasomatic transformations in both the granitic outshoots (greisens) and adjacent or overlying carbonate units (skarns). Mineralization comprises pyrrhotite, pyrite, molybdenite, chalcopyrite, scheelite, wolframite, and gold, as observed in deposits in the northeastern part of the Tichka intrusion (Fig. 2A). In the thermal halo of this pluton, mineralization manifests as Cu–Pb–Zn–Ba with quartz and carbonate gangue^50^.

*Vein mineralization* is observed in structures formed tectonically during the Triassic/Lias extension. The associated magmatism is responsible for fluid circulation, leading to the deposition of Pb, Zn, Cu, and Ba within faults (Fig. 3). Noteworthy examples include the barite veins of the High Seksaoua deposits (Moulay, Ifri, Bouchira, Tassa, and Mtilli), Talat n'Tiouia, Tirquine, and the copper veins of the Taourirt deposit in the Ounein basin^25^. NE- and E- trending faults related to the Atlasic deformation have been identified in satellite images and extend over several tens or hundreds of meters with a width of over two meters.

## Magnetic data and processing

### Data

The aeromagnetic data for this study were collected by the "Geoterrex-Dighem" Canadian geophysical campaign in 1999, under the authority of Morocco’s Ministry of Energy and Mines. These surveys utilized caesium magnetometers with a sensitivity of 0.01nT. The flight lines were oriented N315°, spaced 0.5 km apart, and at an average flying altitude of 30 m above ground to ensure a high-resolution aeromagnetic dataset. These data underwent several corrections to enhance quality, including (i) noise removal (denoising), (ii) elimination of closure errors, and (iii) corrections for diurnal magnetic variations. After applying these corrections, the International Geomagnetic Reference Field (IGRF) was subtracted from the data to isolate the anomalous magnetic field. The resulting total magnetic intensity (TMI) anomaly data were then processed using a gridding cell size of 1000 × 1500 m to create a detailed magnetic anomaly map, providing a crucial foundation for subsequent geological interpretation.

### Processing of magnetic data

**Figure 4:**
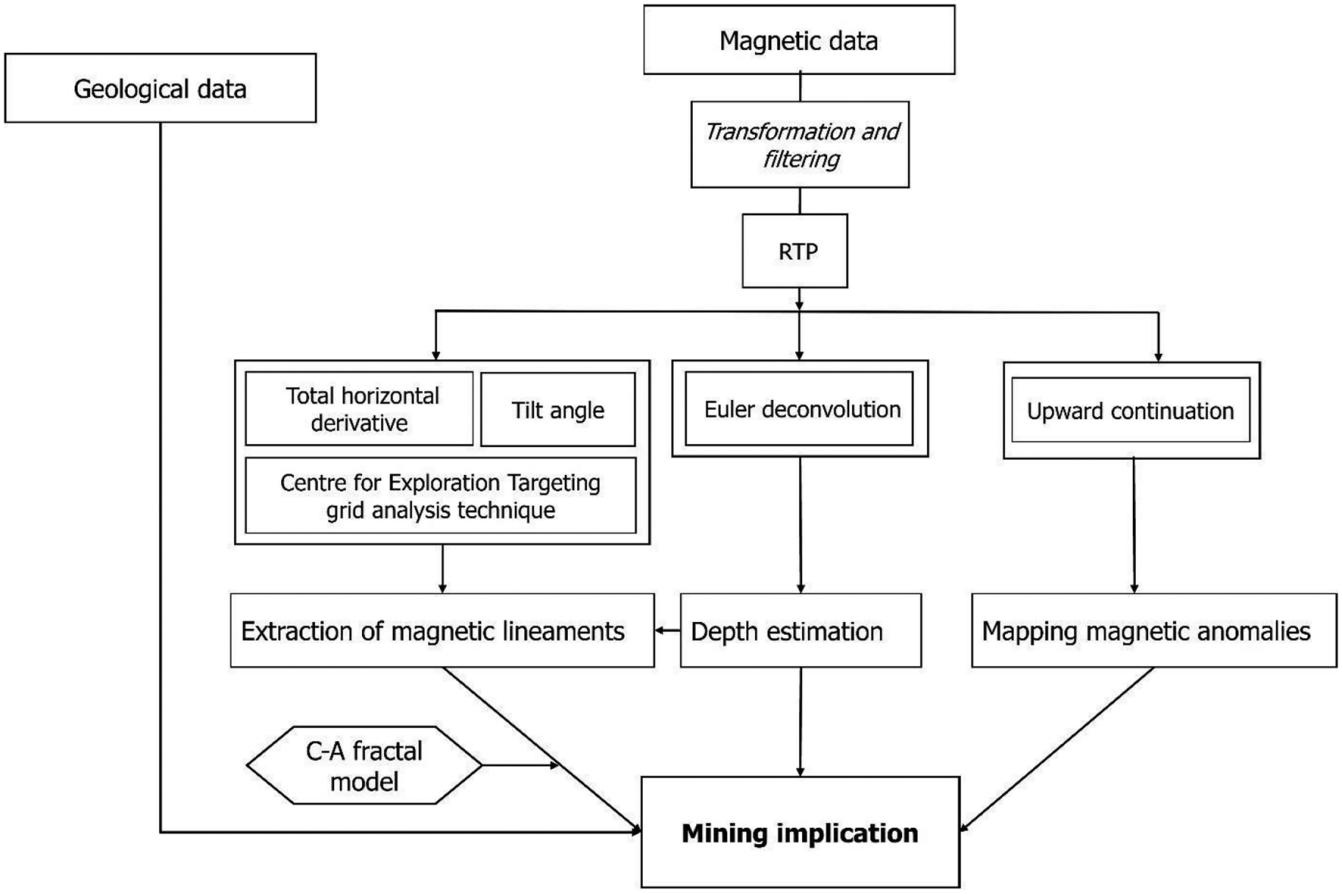
Magnetic Data Interpretation Flowchart. This diagram outlines the step-by-step process used to analyse and interpret the magnetic data, from initial data acquisition to the final geological interpretations.

Magnetic field maps, scaled at 1:50,000, underwent a transition from scanned to georeferenced and digitized formats. This digital transformation facilitated the creation of a comprehensive database, encompassing coordinates and magnetic values, which laid the groundwork for subsequent in-depth data processing and analysis. The analysis of magnetic data proceeded through a series of methodical steps, as illustrated in Fig. 4: (1) Application of the Reduction to the Pole (RTP) procedure on magnetic data to derive the Total Magnetic Field Intensity (TMI), transforming anomalies into symmetric patterns around their causative sources and mitigating the effect of crustal magnetism; (2) Filtering of TMI to differentiate between regional magnetic trends and localized residual magnetic intensity, enhancing the latter for detailed scrutiny; (3) Implementation of separation and enhancement filters for lithological and structural mapping, such as upward continuation and total horizontal derivative, tilt angle, and Euler deconvolution, through grid analysis. This process aimed at mapping structural complexity and interpreting transformed data to identify anomalies, magnetic lineaments, and develop a comprehensive structural scheme; (4) Finally, generating a lineament density map using the C-A method, classifying it, and integrating it with structural data and known metallogenic indices to delineate areas likely to host tectonically controlled mineralization.

These steps ensured a meticulous processing of magnetic data, leading to the delineation of significant geological and structural insights pertinent to our study's objectives.

#### Reduced-to-the-pole magnetic and upward continuation.

**Figure 5:**
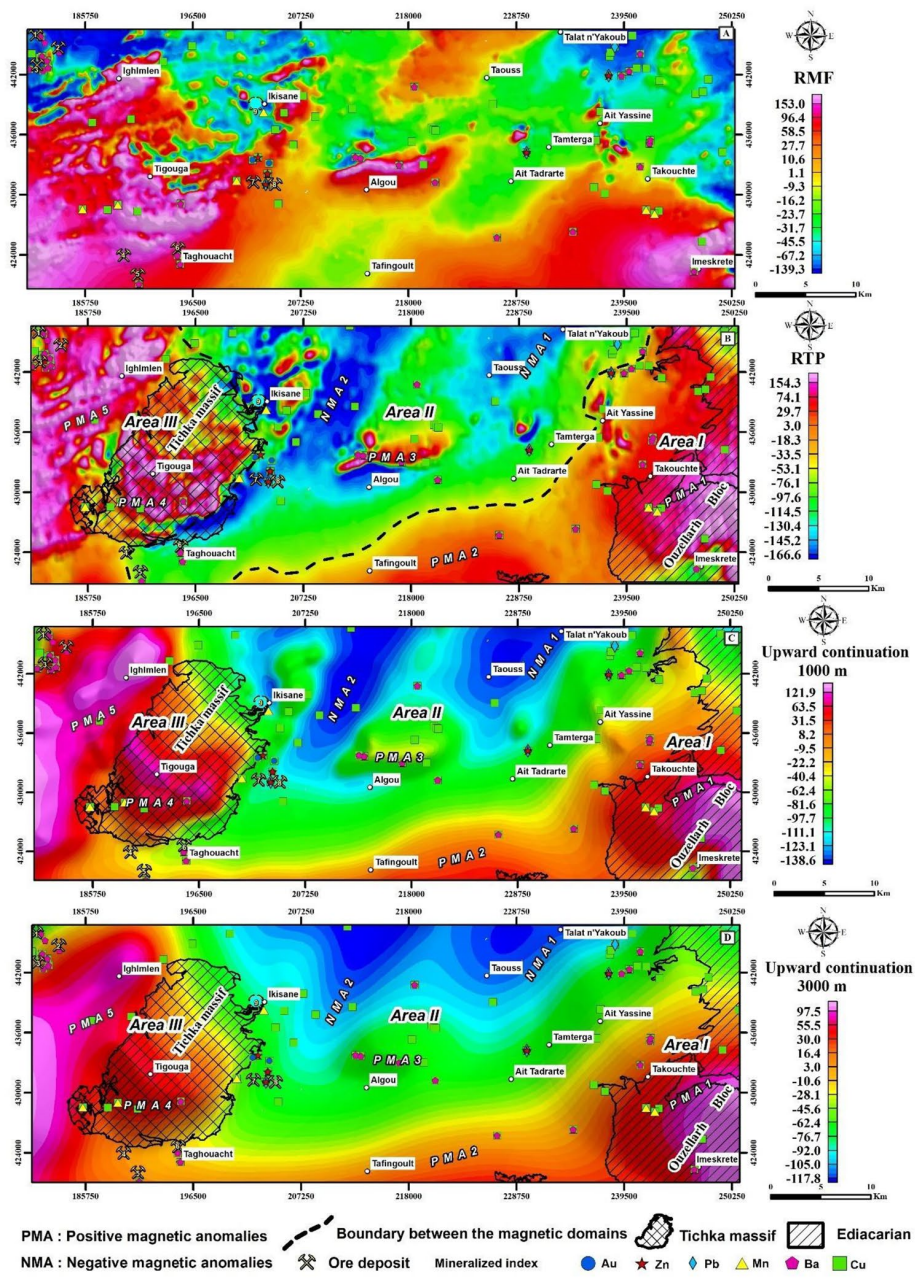
Comprehensive Analysis of Aeromagnetic Data: (**A**) Residual Magnetic Field (RMF) anomaly map, obtained after subtracting the IGRF from the measured data to isolate the Total Magnetic Intensity (TMI) anomalies. (**B**) Reduction to the Pole (RTP) magnetic anomaly map. (**C**) and (**D**) Illustrate the RTP data after upward continuation to 1000 m and 3000 m, respectively, to examine anomaly trends at different crustal levels. The maps were created using Geosoft Oasis Montaj software, version 2023.2.0.28 (https://www.seequent.com/products-solutions/geosoft-oasis-montaj/).

The RTP transformation was calculated using a declination of − 4.5° and inclination of 41.9°, corresponding to the IGRF parameters on January 1, 1999^51^. The RTP transformation positions magnetic anomalies directly above their causative sources, making interpretation easier and more reliable^51^. The subsequent RTP magnetic anomaly map is shown in Fig. 5B.

Following RTP correction, an upward continuation was applied to the magnetic data at altitudes of 1000 and 3000 m (Fig. 5C,D), delineating magnetic sources at depths beyond 500 and 1500 m, respectively, thereby enhancing the identification of magnetic anomalies and aiding in the geological interpretation of subsurface structures^32^. The filter was applied to attenuate short-wavelength anomalies^52^. This method has several advantages, including removing high-frequency spatial noise and highlighting regional magnetic anomalies. The resulting maps illustrate the distribution of magnetic susceptibility sources, highlighting those at different depths that are particularly significant. The representation aims to delineate the depths of magnetic sources, with each map focusing on different depth ranges to provide a comprehensive understanding of subsurface magnetic anomalies.

#### Extraction of magnetic lineaments using derivative methods

Derivative methods, such as the Total Horizontal Derivative and Tilt Angle, alongside grid analyses from the Centre for Exploration Targeting^12^, were utilized to delineate magnetic lineaments associated with lithological contacts, faults, and crustal lithological discontinuities^53^. These methods were used to analyze the RTP data to determine the linear magnetic anomalies and relate them to structures controlling mineralization hosted within different lithological units in the study area. The magnetic lineaments were categorized into two categories: major lineaments, associated with major block boundaries and/or shear zones, and minor lineaments, associated with minor linear segments. Two rose diagrams were produced showing the directions of major and minor magnetic lineaments.

**Figure 6:**
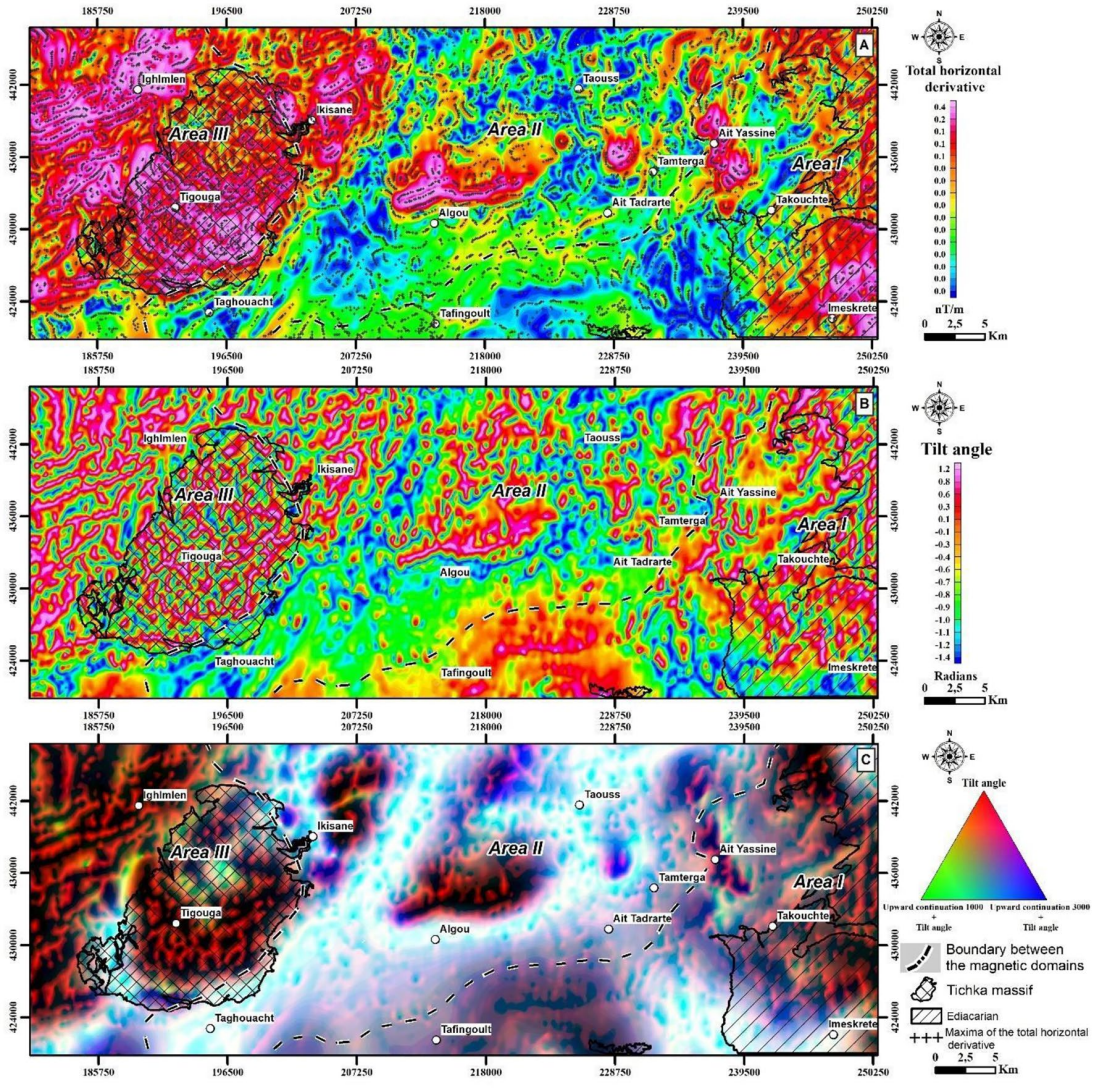
(**A**) The Total horizontal derivative operator applied to the RTP data. (**B**) Tilt angle map of the RTP data. (**C**) Features a Ternary image combining Tilt Angle data (red) with Tilt Angle from the 1000 m (green), and 3000 m (blue) Upward Continued (UC) maps, offering a comprehensive visualization of magnetic anomalies at different depths. The maps were developed using Geosoft Oasis Montaj software, version 2023.2.0.28.

The total Horizontal Derivative (THD) method calculates the vector sum of a magnetic field’s horizontal derivatives to emphasize lateral field variations and attenuates broad regional trends^8^. This approach is particularly effective in areas with pronounced magnetic susceptibility differences, highlighting the edges or discontinuities of geological structures. The formula for THD (Eq. 1), involving the square root of the sum of squared derivatives in both x and y directions, serves to highlight the boundaries of geological features, especially where these features have steep or shallow inclinations. The total horizontal derivative map of the RTP map is shown in Fig. 6A.

1 $${THD}_{\left(x,y\right)}=\sqrt{{\left(\frac{\partial f}{\partial x}\right)}^{2}+{\left(\frac{\partial f}{\partial y}\right)}^{2}}$$

The Tilt angle method (Eq. 2), an analytical tool for structural analysis and depth estimation of geological features. It calculates the angle between the total horizontal derivative^8^ and the vertical derivative^54,55^ of the magnetic field. This filter is useful in delineating subsurface geological structures^55^ and estimating the depth to causative sources^56^. The tilt angle values, which range from − 90° to + 90° degrees, generate contours that closely correlate with the proximity and depth of magnetic sources. These contours facilitate precise geological and structural interpretations based on their spatial distribution. Specifically, it produces zero-value contours at the contact with magnetic sources^10^, positive-value contours located above these sources, and negative-value contours located away from the magnetic sources. The method also utilizes the distance from the zero contour to the contour at a tilt angle of π/4 (or 0.78540 radians), offering an approximation of the depth of the underlying contact. This approach assumes that the tan(π/4) = 1, where the horizontal distance (h) to the vertical distance (d) ratio equals one, facilitating the depth estimation of magnetic bodies beneath the magnetometer. The Tilt angle is shown in Fig. 6B.

2 $${TDR}_{\left(x,y\right)}={tan}^{-1}\left[ \frac{\frac{\partial f}{\partial z}}{\sqrt{{\left(\frac{\partial f}{\partial x}\right)}^{2}+{\left(\frac{\partial f}{\partial y}\right)}^{2}}}\right]$$

**Figure 7:**
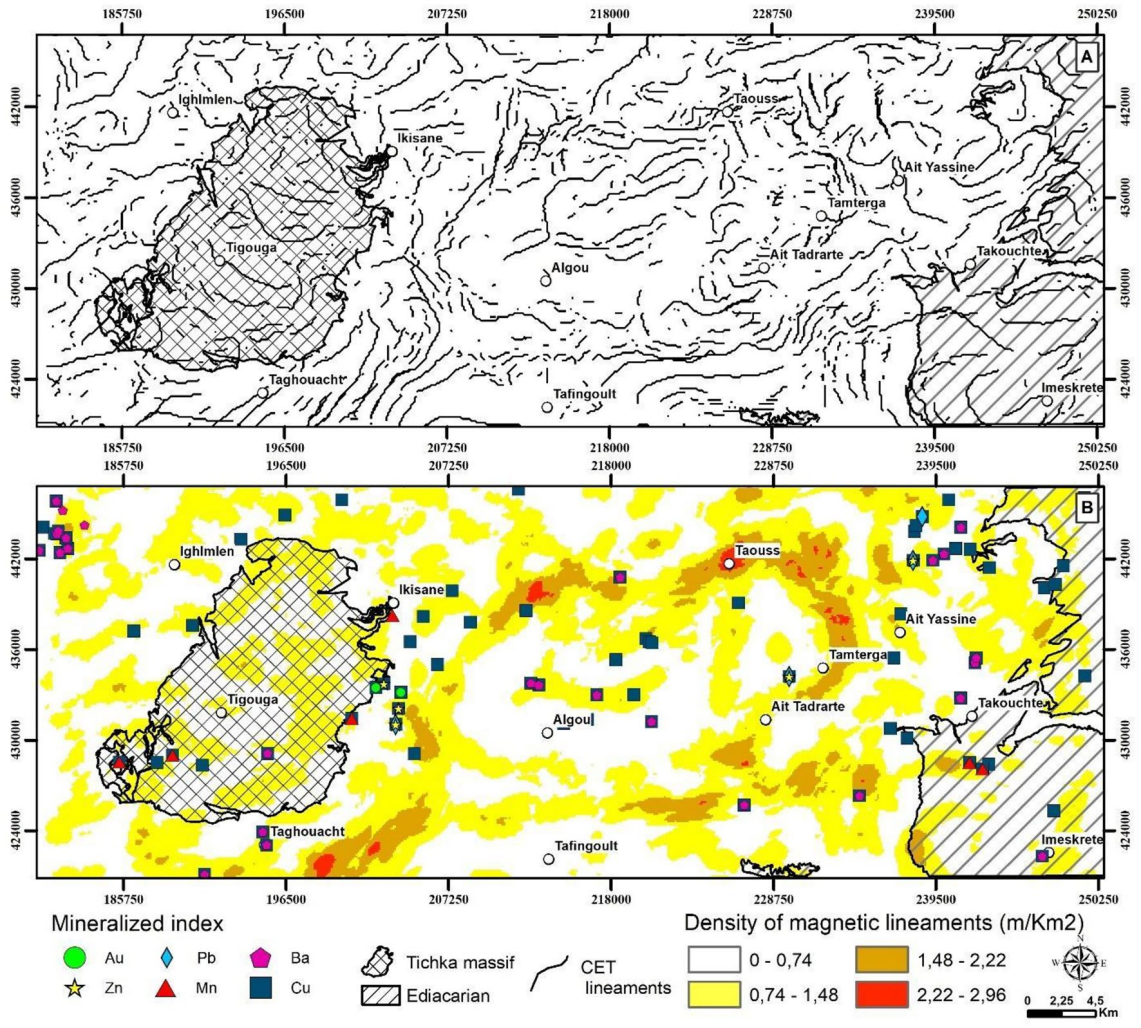
Exploration Targeting and Lineament Analysis. (**A**) Displays the Centre for Exploration Targeting (CET) map, showing the derived faults crucial for mineral exploration. (**B**) Illustrates a Magnetic Lineament Density map, derived from the CET grid analysis, to visualize areas with high concentrations of magnetic lineaments indicative of potential exploration targets. The maps were created using ArcGIS, version 10.6.1.

A Centre for Exploration Targeting grid analysis^12^, uses phase congruence where discontinuities and linear features in a dataset all have components that are maximally in phase after a 2-D Fourier transformation, whether one is looking for symmetric or asymmetric features. The Centre for Exploration Targeting analysis has the advantage of providing a dimensionless value (phase congruency in the Fourier-transformed frequency domain) that is independent of amplitudes or contrast in the original data. A Centre for Exploration Targeting grid analysis begins with a texture analysis which is an image enhancement method that looks for subdued magnetic responses. The texture analysis will first enhance the local data contrast and is followed by structure detection (phase congruency) which is useful for identifying linear discontinuities. The discontinuity structure detection takes the texture analysis output and builds a skeletal structure of the regions of discontinuity. The output is a set of binary line segments separating each of the discontinuity regions, isolating orientation changes and offsets caused by discrete magnetic susceptibility structures. A final step aims to vectorize the linear segments by examining the texture peaks identified through phase symmetry. Magnetic discontinuities can reveal lithological boundaries, faults, and dykes and are helpful to understand local and regional geology. Additionally, the mapped structures can be used to locate areas favourable for mineral exploration^12^. Figure 7 illustrates the outcomes of the Centre for Exploration Targeting grid analysis.

#### Depth estimation

Euler deconvolution^13^, utilized on RTP- upward continuation magnetic data, aids the localization and depth estimation of magnetic sources. This method relies on Euler's homogeneity equation (Eq. 3)^13^, which can be written as follows:

3 $$\left(x-{x}_{0}\right)\frac{\partial f}{\partial x}+\left(y-{y}_{0}\right)\frac{\partial f}{\partial y}+\left(z-{z}_{0}\right)\frac{\partial f}{\partial z}=SI\left(B-f\right)$$

where (× 0, y0, z0) in (3) represents the position of the magnetic source, (x, y, z) represents the position of the observation point, f represents the measured total field at (x, y, z), B represents the regional field component contained in the total anomaly^13,57^, and (SI), represents the degree of homogeneity.

**Figure 8:**
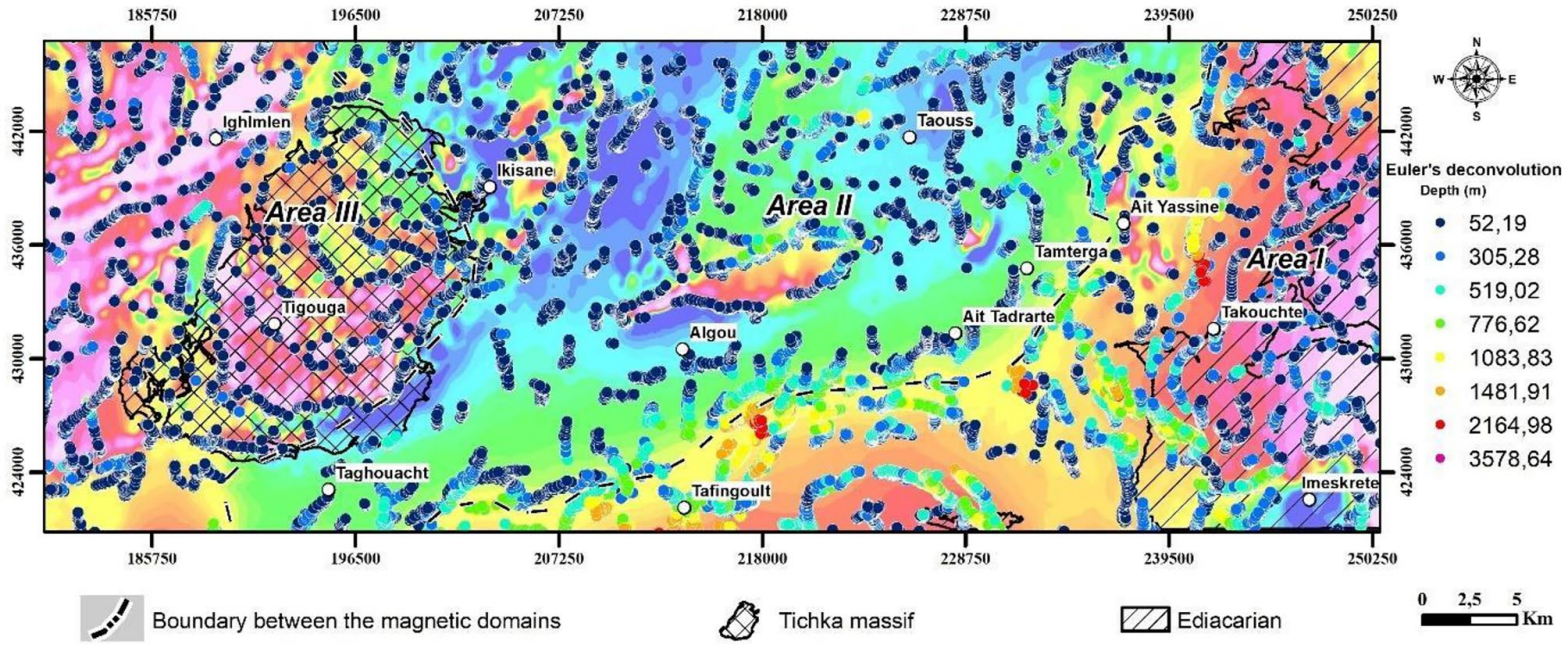
Depth Estimation via Euler Deconvolution. This Fig presents the depths to magnetic sources within the study area, determined using Euler deconvolution applied to the RTP data with a structural index (SI) of 0, highlighting the variability and distribution of subsurface features. The maps were created using Geosoft Oasis Montaj software, version 2023.2.0.28.

Accuracy in depth estimations is influenced by factors like structural index (SI), window size, grid spacing, and the spacing of collected data. Importantly, grid spacing should match the collected data's spacing^58^. First, the data grid spacing must reflect the actual data spacing. Second, the choice of the window size when applying the Euler deconvolution equations will determine the maximum depths as Reid et al. in 1990^57^ showed that the maximum depths correspond to roughly twice the window size. Additionally, the window size should be large enough to include significant variations in the field and its gradients yet small enough to avoid including the effects of multiple sources^59^. The chosen window size of 12 km typically yielded maximum depths between 8 and 10 km. Third, the SI must be chosen to represent a model for the source geometry and is based on the local geology. We determined (SI = 0), which represents a pole line (e.g., a contact), best represented the geometries of the sources in the study area. Figure 8 shows the results of the Euler Deconvolution analysis.

#### Modeling magnetic lineaments using C-A multifractal model

The concept of fractals, initially introduced by^60^ has found extensive application in the geosciences for generating maps that depict the spatial distribution of classified patterns. These maps are instrumental in mineral prospectively modeling. However, conventional fractal models, which are based on a single component, often fall short of accurately representing complex systems. To address this limitation, multifractal models were developed, enabling the exploration of such systems through a continuous spectrum of exponents or fractal dimensions^61^. Additionally, a multifractal model provides more information about measurements on spatial objects than a fractal model*.* There are several types of fractal models including Number-Size, Concentration-Area (C-A), Spectrum-Area, Concentration-Concentration, Concentration-Distance from Centroid, Concentration-Volume and Power Spectrum Volume^61^. Multifractal modeling theory includes several models^62–64^. The (C–A) multifractal model^65^, based on equation A (ρ ≤ v) ∝ ρ − α1A (ρ ≥ v) ∝ ρ − α23, was used in this study to determine thresholds representing different groups with varying degrees of importance in controlling mineralization through faults^66^. A(ρ) represents the surface values of concentration greater than or equal to the contour value ρ (the value of each pixel in a geospatial image), α1 and α2 represent the characteristic minimum and maximum exponents, and v is the cutoff point or threshold. This latter parameter can be determined in the model by determining the fractal dimension from the intersection point of several classes^67^.

## Data analysis

### Lithological analysis

Analysis of the (Fig. 5A) reveals several magnetic anomalies of different wavelengths and amplitudes, which correlate directly with the lithological nature of the geological formations prospected. These anomalies reflect the signal intensities among facies. Through the RTP map analysis, marked by contrasting anomalies, we categorize the region into three magnetic domains (Fig. 5B). The first domain (**Area I**) in the southeastern part of the area covers the entire Ouzellarh Block, displaying long wavelength positive magnetic anomalies with intensities ranging from 49 to 199 nT. These anomalies are related to the sub-basement’s composition, primarily ignimbrite nappes episodically interspersed with andesitic and rhyolitic lavas of the Ediacaran age, known for their high magnetic mineral content. This composition is particularly evident in the southeast, where the highest amplitude maxima (PMA1) is located. Moving west and southwest, where the Cambrian sedimentary cover overlays the basement, we observe anomalies PMA1 and PMA2. Here the Cambrian sediments, folded into the NW–SE trending Adrar Doutganza syncline during the Variscan orogeny, cover the basement. Despite the prevalence of Anti-Atlas aged formations, indicating Precambrian block uplifts and disharmonic folding of the Paleozoic cover^68^, these structural features primarily affect the basal series at the basement level rather than the overlying formations. However, the relative thinness of this cover (< 1000 m), coupled with the shallow depth of the Ediacaran basement, results in persistent magnetic maxima due to the abundance of magnetic minerals within the basement lithologies. **Area II** is characterized by smaller amplitude magnetic anomalies, including two NNE-trending elongated magnetic minima zones (NMA1 and NMA2). These minima align with fault direction tha influenced the Lower Cambrian graben formation^46^, reflecting thick Paleozoic deposits exceeding 5000 m in depth within NNE-trending collapsed trenches. A notable magnetic maximum (PMA3), situated north of Algou, is attributed to Tichka granitic intrusions, potentially augmented by nearby shear zones. The demarcation between Area I, and Area II, coincides with the Ouchden Fault, delineating the distinction between the deep western Mesetian graben (Areas II and III) and the shallow eastern anti-Atlas graben (Area I). **Area III**, located in the western study area, characterized by high amplitude maxima (PMA4) linked to the presence of a large granitoid intrusion within the Tichka massif, incorporating gabbroic rocks, quartz diorites and granodiorites. In addition, the Lower Cambrian within this area is rich in volcanic and volcaniclastic facies, marked by short wavelength, high amplitude magnetic maxima (PMA5) likely associated with magnetic material along the major faults. Upward continuation of RTP data to altitudes of 1000 m and 3000 m, as shown in (Fig. 5C,D), reveals that anomalies persist at a 1000 m depth but diminish at 3000 m, suggesting the sources are shallower than 1500 m. This observation, particularly with anomaly PMA3, implies that the anomalies likely originate from shear zones rather than magmatic intrusions.

Upon analysis, the persistence of magnetic anomalies at a depth of 1000 m, coupled with their diminishment at 3000 m, suggests the presence of features consistent with shear zones. This pattern contrasts with the expectations for magmatic intrusions, which would likely exhibit a more uniform magnetic response due to their coherent volume. The inferred shear zones, characterized by fractured or altered rock, can significantly impact the magnetic signature at varying depths, supporting our interpretation of the subsurface geological structures.

The magnetic anomaly along the Ouchden Fault, persisting over 15 km, marking the boundary between two magnetically contrasting domains. Area I, with its magnetic maxima, aligns with the rigid Precambrian block of Ouezllarh, contrasting with the Area II’s magnetic minima domain, reflective of the western Paleozoic block of the HA. Notable field observations include a variable dip angle reverse fault, with an estimated displacement of 1000 m^25^, hinting at the complexity of fault dynamics within these zones, including potential thrust fault characteristics, as evidenced 10 km southeast of Tamterga.

### Structural analysis and depth estimation

The application of total horizontal derivative and Tilt angle filters to the RTP data (Fig. 6A,B) has been revealed distinctive lineaments in the study area. In Area I, multiple linear anomalies were observed, whereas Area II displayed NNE trending linear anomalies near a granitic intrusion contact (Tichka granite), alongside ENE-WSW and NE-SW trending lineaments central to the study area and at the boundary between Areas I and II, respectively. Area III exhibited high-amplitude magnetic derivatives, as shown in Fig. 6B, associated with the Tichka intrusive complex (identified as PMA 4 in Fig. 5) and linear anomalies trending NE and NNE along faults identified as PMA 5 in Fig. 5.

To emphasize the depth continuity of the lineaments, results from three different Tilt angle filters (Tilt angle, upward continuation 1000 + Tilt angle and upward continuation 3000 + Tilt angle) were combined into an RGB format (Fig. 6C). This allowed the classification of lineaments into major and minor sets based on their wavelengths^69^. Euler Deconvolution further provided depth estimations for magnetic susceptibility sources, and the depth solutions (Fig. 7) with color-coded solutions indicating most sources are shallower than 500 m (Fig. 8), particularly in Areas II and III. Depths in the Paleozoic–Mesozoic sedimentary cover (PMA 2) in OB, depths exceeded 4000 m, suggesting deeper lineaments than surface geological maps show (Fig. 5A). This investigation confirms the Anti-Atlas’s structural complexity due to Variscan inversion, characterized by block tectonics and disharmonic folding of the Paleozoic cover^70^.

The Centre for Exploration Targeting grid analysis effectively delineates linear magnetic regions and zones of structural complexity within the study area, as illustrated in Fig. 7A. This analysis, exemplified through a density map of lineaments (Fig. 7B), indicates the area’s complex structure, characterized by various orientations of magnetic discontinuities. These magnetic discontinuities, potentially representing dykes, contacts, faults and shear zones, predominantly align along NE-SW to E-W oriented deformation corridors, including notable zones like the Tizi n'Test, the Arg and the Ouchden fault zones. A notable correlation exists between these deformation corridors and mineral occurrences, with high lineament density areas often coinciding with mineralized locations. This suggests that areas with dense lineaments could be prime targets for mineral exploration, indicating prospective zones for mineral deposit emplacement.

### Analysis of the lineament map classified by fractal modeling

**Figure 9:**
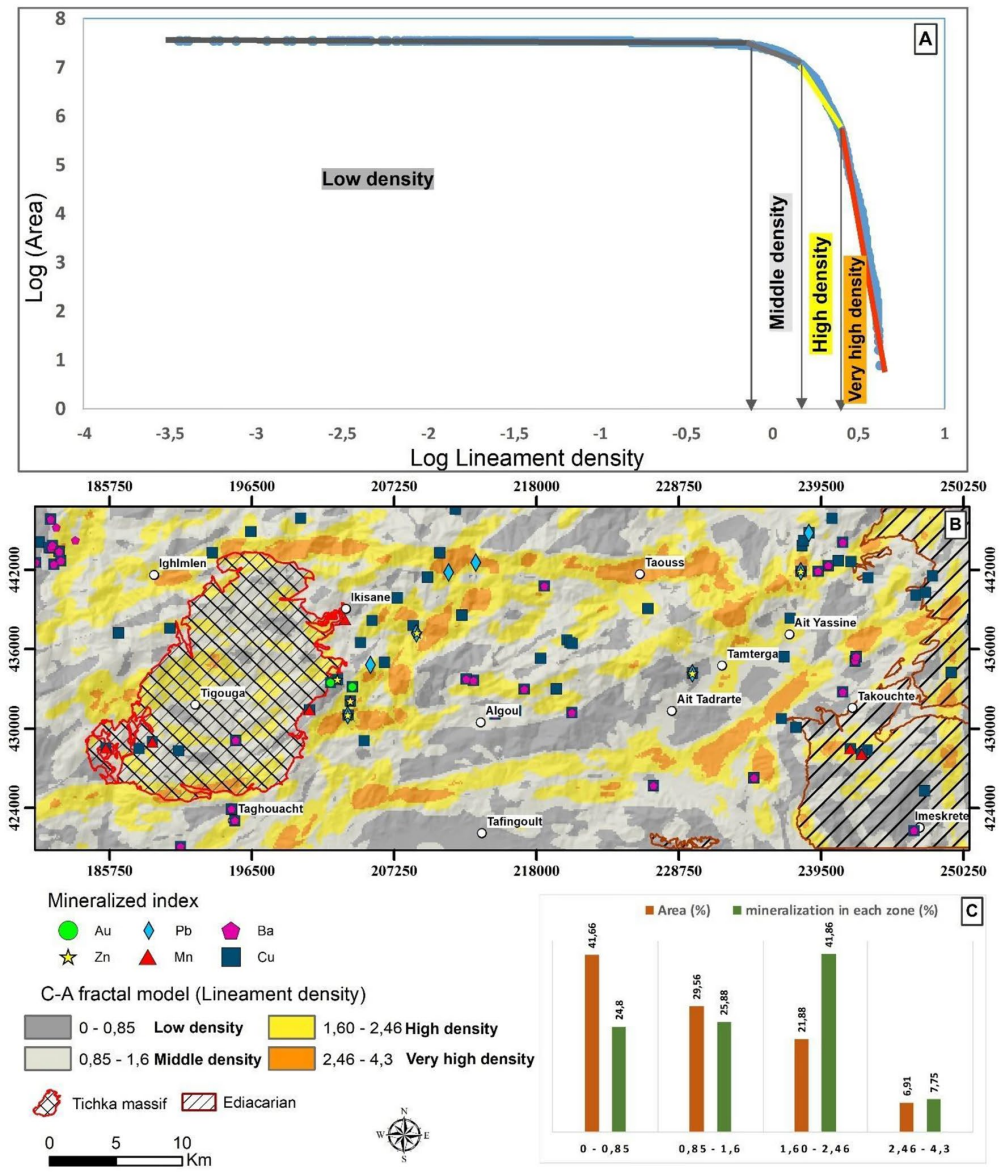
Fractal Analysis of Magnetic Lineaments. (**A**) Illustrates the Concentration–Area (C-A) fractal model applied to analyse magnetic lineament distributions. (**B**) Classifies lineaments into distinct groups based on the C-A fractal model, demonstrating the methodology for categorizing geological features. (**C**) Shows the distribution of metal references in each class, providing insights into the correlation between fractal analysis results and known metal occurrences. The maps were generated using ArcGIS, version 10.6.1.

The classification of magnetic lineaments through the C-A multifractal model (Fig. 9A) has leveraged a log–log plot of the lineament density map (Fig. 9B), revealing a consistent slope indicative of a fractal dimensionality. Breakpoints on the plot served as thresholds for classifying the magnetic lineament density map, effectively visualizing zones with a preference for structurally related mineralization.

Statistical analysis of fractal dimensions (Fig. 9C) distinguished four populations based on lineament density. The first two, with low to medium lineament densities, constitute 41.66% and 29.65% of the total study area, hosting 50.68% of all outcropping mineral occurrences. Conversely, the last two, characterised by high lineament densities, cover 28.79% of the total area. Despite their smaller spatial coverage, these populations encapsulate 49.51% of the outcropping mineral occurrences. This statistical examination provides valuable insights into the relationship between lineament density and mineralization occurrence within the study area.

## Discussion

### Structural implication

**Figure 10:**
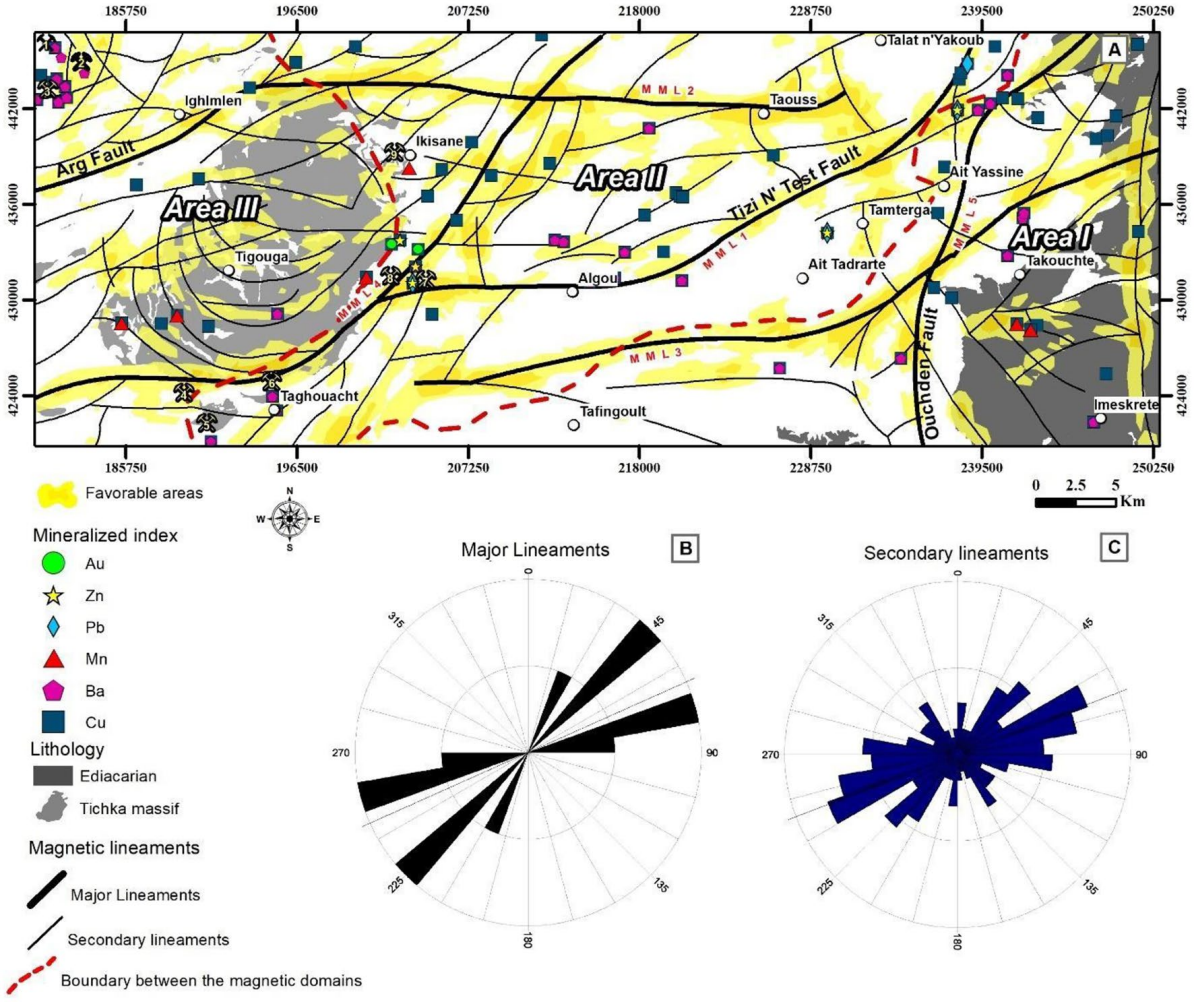
Analysis of Structural Elements and Lineament Orientations (**A**) Structural elements within the study area determined from the magnetic data analysis, listing significant locations such as Tirquine, Talat n’Tiouia, Ifri n’Janjar, Goudacha, Jbel Boufounas, Taghouacht, Jeralna, Alebdi, and Ikisane. (**B**) and (**C**) Present Rose diagrams showing the predominant orientation of major and secondary lineaments, respectively, offering insights into the structural directions influencing mineralization and geological processes in the region. The maps were produced using ArcGIS, version 10.6.1.

In the comprehensive analysis and refinement of magnetic data, numerous magnetic susceptibility discontinuities were identified, hinting at potential associations with the structural evolution of the study area. The resultant magnetically derived structural map of the Tizi n'Test region (Fig. 10A) offers a new perspective on the regional tectonics of the study area. The orientations of magnetically derived lineaments are illustrated in rose diagrams (Fig. 10B,C). revealing predominant trends along the NE-SW, ENE-WSW, and E-W directions, with additional trends noted in NW–SE and N-S directions (Fig. 10C). Structurally, these magnetic lineaments closely align with major structural trends arising from diverse orogenic and extensional tectonic episodes in the High Atlas.

**Figure 11:**
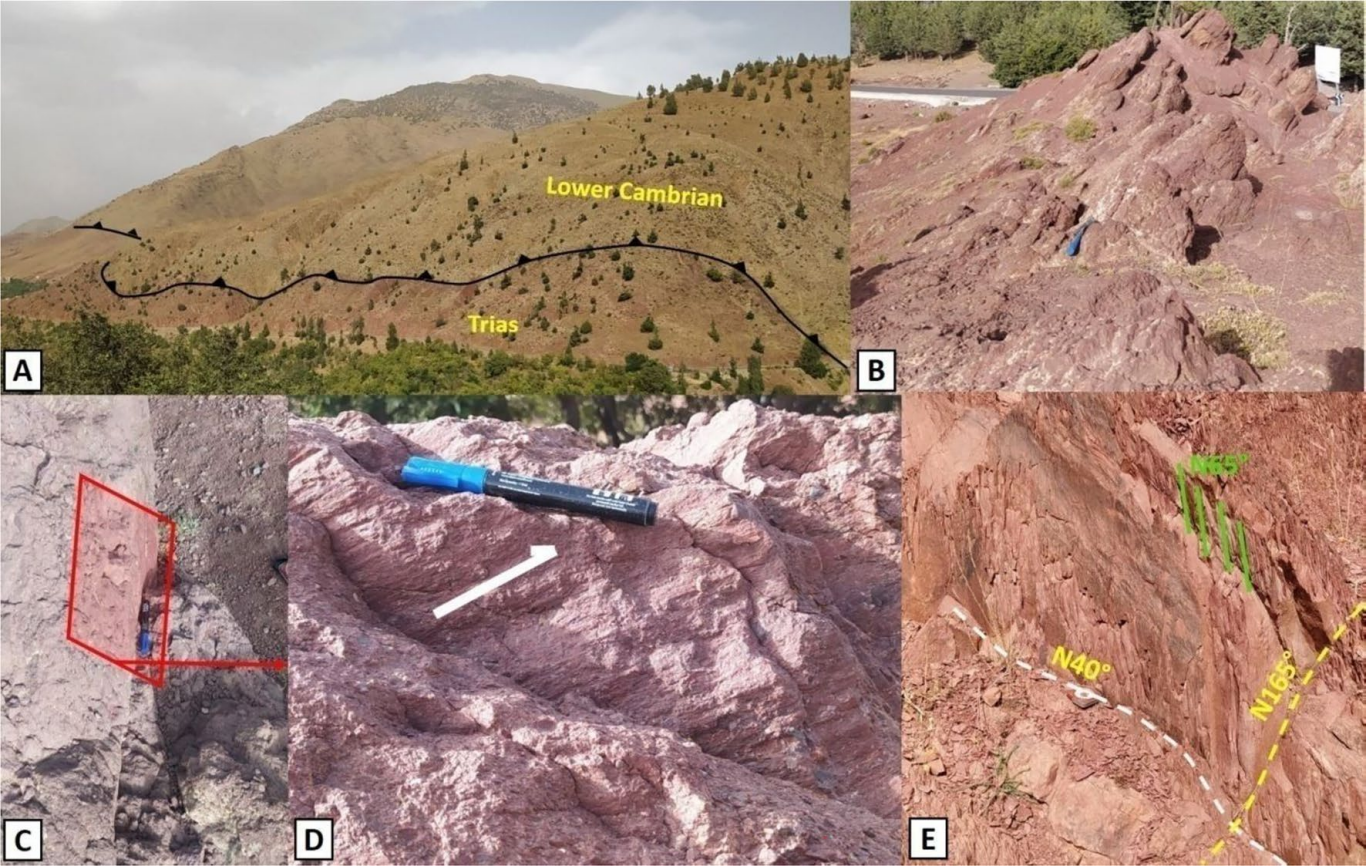
Tectonic structures mapped along the Tizi n Test fault zone. Highlights critical tectonic features observed: (**A**) Triassic overlapped the Lower Cambrian. (**B**) E-W reverse fault affecting the Permian conglomerates. (**C**,**D**) E-W sinistral strike-slip fault. (**E**) Fractures (N40° and N165°) and schistosity (N65°) in the Triassic clays, illustrating the complex tectonic interactions within this fault zone.

**Figure 12:**
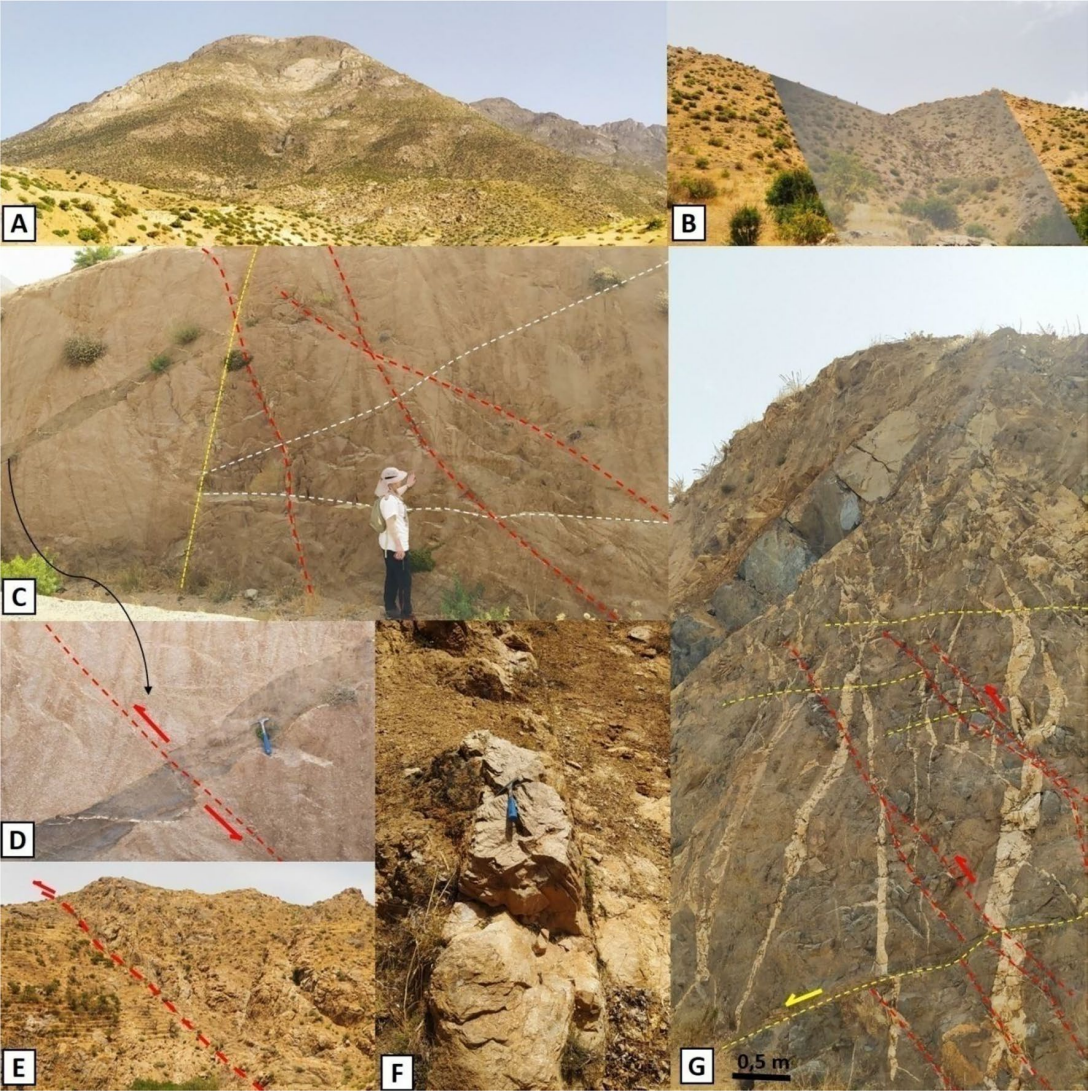
Main directions of the brittle structures mapped in the Tichka massif. (**A**) Zebra aspect of the Tichka massif. (**B**) N-S oriented fault in the granite. (**C**) Clear contact between diorite and granodiorite (granodiorite appears in clear) and NE-SW to E-W directions affect this complex. (**D**) dolerite vein offset by reverse faulting. (**E**) Lower Cambrian to Middle Cambrian thrusting. (**F**) aplitic vein intrusive in granodiorite. (**G**) Quartz veins offset by reverse faulting.

The NE-SW to E-W-oriented lineaments, encompassing major structures MML1, MML2, and MML3, alongside minor lineaments, emerge as the most prevalent trends in terms of frequency (Figs. 10A–C, and 11). These trends correspond to the Tizi n'Test fault zone (MML1) and the Arg fault zone (MML2). Kinematically, these extensive fault areas have undergone multiple tectonic events, including vertical movements in the Lower Paleozoic^71^, influencing the formation of a Cambrian basin, and crustal shortening at the end of the Carboniferous^20,43,72^ due to the Variscan orogeny. From the Permian to the Triassic, the region underwent extensional tectonics, forming grabens or semi-grabens bounded by active faults (Fig. 11A). In addition, the late Hercynian structures were reactivated during the Alpine orogeny (Fig. 11C,D), and additional compressive tectonic structures were formed during the Mio-Pliocene (Fig. 11B). Except for the E-W trending faults and the thrusts faults, most of the faults are typical of those formed during the Variscan orogeny and some have replayed during the Atlas orogeny. Some faults are injected by veins (basic or acidic) that have remained open and do not appear to have replayed since their emplacement (Fig. 12A–G).

The NNE-SSW-oriented magnetically derived lineaments (Fig. 10B,C) represented by MML4 and MML5 are visible on magnetic maps (Figs. 6C and 7A). These lineaments border the Tichka granite on its eastern part and the OB on its western part. These lineaments may be related to structures that were reactivated during the post-Pan African extension, giving rise to the opening of the NNE-oriented basins during the Lower Cambrian rifting stage^46^, This opening is coincides with the opening of the Rheic Ocean^40^. Two distinctive styles of deformation characterize the tectonic regime in the study area. One style is similar to that found in the Anti-Atlas in which the Hercynian deformation reactivated faults inherited from basement structures formed during the Neoproterozoic/Cambrian rift event. These reactivated faults induced vertical motions of the Precambrian blocks^73^. The second style resembles the structural styles found in the Meseta where synschist deformation occurred in the western block of the HA. Magnetically, the first style is well known in the magnetic Area I corresponding to the OB, while the second is located in Areas II and III attributed to the HA. MML3 and MML5 separate the two domains (AMHA and OB). The synthesis of previous works^38,39,45,74,75^ shows that the Tizi n’Test fault zone constitutes a barrier that separates the two domains (Area I and Area II). However, our magnetic analysis leads us to suggest that the Ouchden fault (MML5) (Fig. 10A) acts as a barrier between the two domains, (Figs. 5B–D, and 6C).

## Implication for mining exploration

### Implication for mining exploration

Mineral deposits formed through hydrothermal processes often localize near faults, discontinuities, or continental margins^15,76,77^ within the Earth's crust. These geological structures are not merely passive witnesses to tectonic movement; they actively influence the orientation, circulation, and concentration of hydrothermal fluids^16^. Consequently, they serve as vital indicators for the exploration and detection of epigenetic mineralizations associated with tectonics in neighbouring rock formations^78^. In the ancient Haut Atlas massif, structural control of mineralizations is a key element, influencing not only their genesis, mobilization, and trapping but also driving their rejuvenation and redistribution (see Section "Mineralization"). This region is marked by several tectonic and magmatic events that have led to the formation of diverse mineralizations (Fig. 3). In this study, integration of geological and geophysical data, notably through the density map of magnetic lineaments established using enhancement filters and Centre for Exploration Targeting grid analysis and classified using fractal modeling C-A (Fig. 10A), allows for identification and mapping of areas potentially favourable for metallic ore deposits. Furthermore, analysis of magnetic texture from RTP maps and upward continuation has enabled the division of the studied area into three distinct magnetic domains. However, due to the relatively large spacing between flight lines, set at 500 m, the magnetic anomalies mapped in these three domains cannot be directly attributed to metallic mineralizations. Instead, they result from variations in the mineralogical composition of rocks, the presence of magnetic minerals, or hydrothermal alterations.

**Figure 13:**
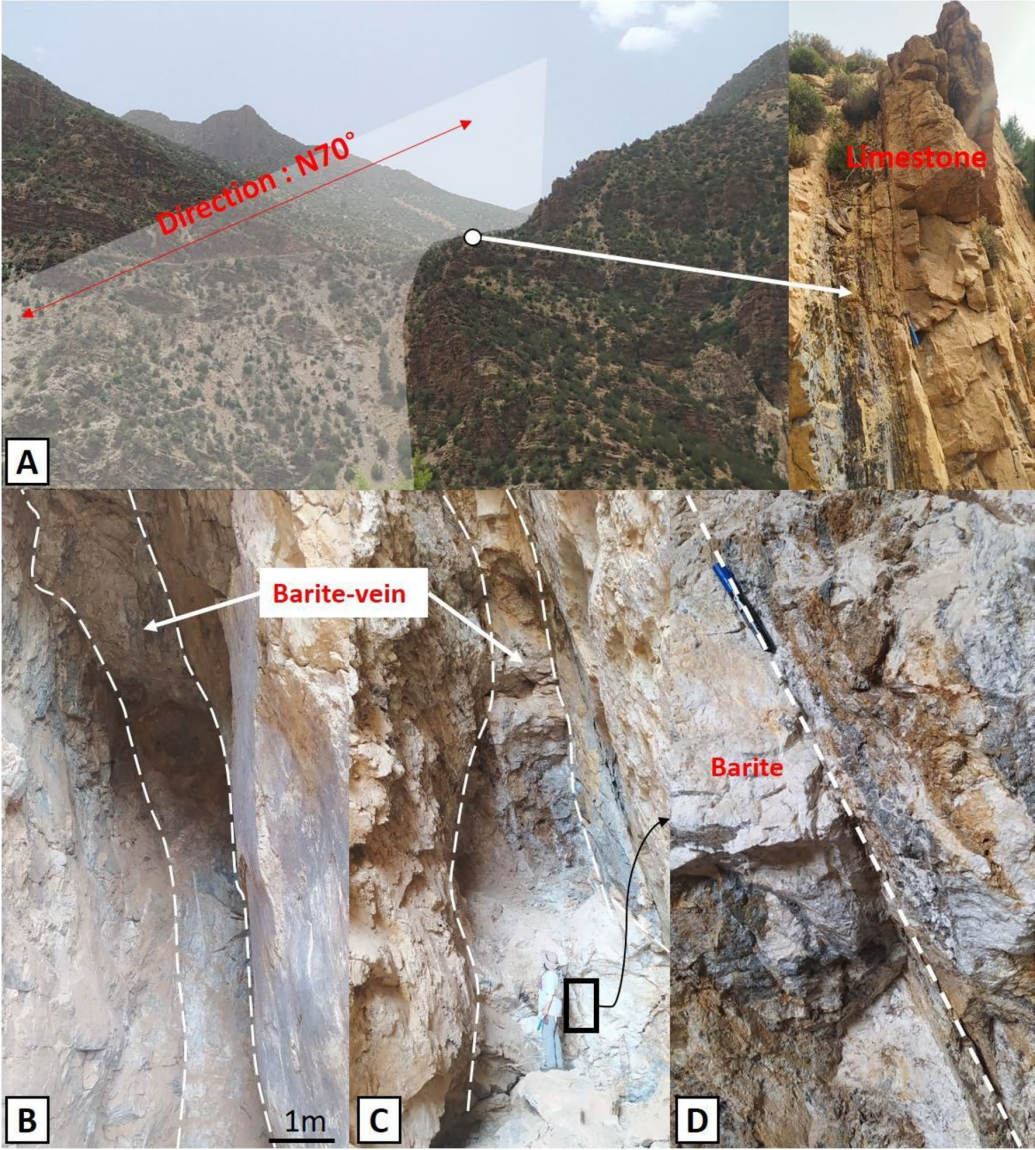
(**A**) Ba(Cu-Pb) mineralization in the Lower Cambrian of OB: N70° regional fault controlling the vein field. (**B**,**C**) Ba ± Cu-Pb veins. (**D**) Late Atlas fractures associated with hydrothermal alteration affecting mineralization.

In Magnetic Area I, copper (Cu), barium (Ba), and lead (Pb) deposits are closely associated with the magnetic contrast zone at the boundary between Magnetic Areas I and II, and is particularly pronounced in the PMA1, corresponding to the volcano-sedimentary formations of the Ouarzazate Group. These formations encompass a variety of facies, ranging from basalts to rhyolites, including basic andesites, andesites, and rhyodacites. It's worth noting that the contact between the basement and carbonate cover constitutes a fertile zone in the Anti-Atlas chain, hosting numerous copper-bearing deposits^16^. Additionally, the structural control of these mineralizations is well defined, with most occurrences aligned along N-S, NNE-SSW, and NNW-SSE oriented lineaments (Fig. 10b, c). These lineaments extend to depths ranging between 200 and 4000 m (Fig. 8). For instance, mineralizations located at the intersection of the Ouchden Fault and a NE-SW oriented lineament, constituting the satellite of the Tizi n Test fault zone between Ait Yassine and Talat n’Yakoub localities, serve as an example (Fig. 13).

**Figure 14:**
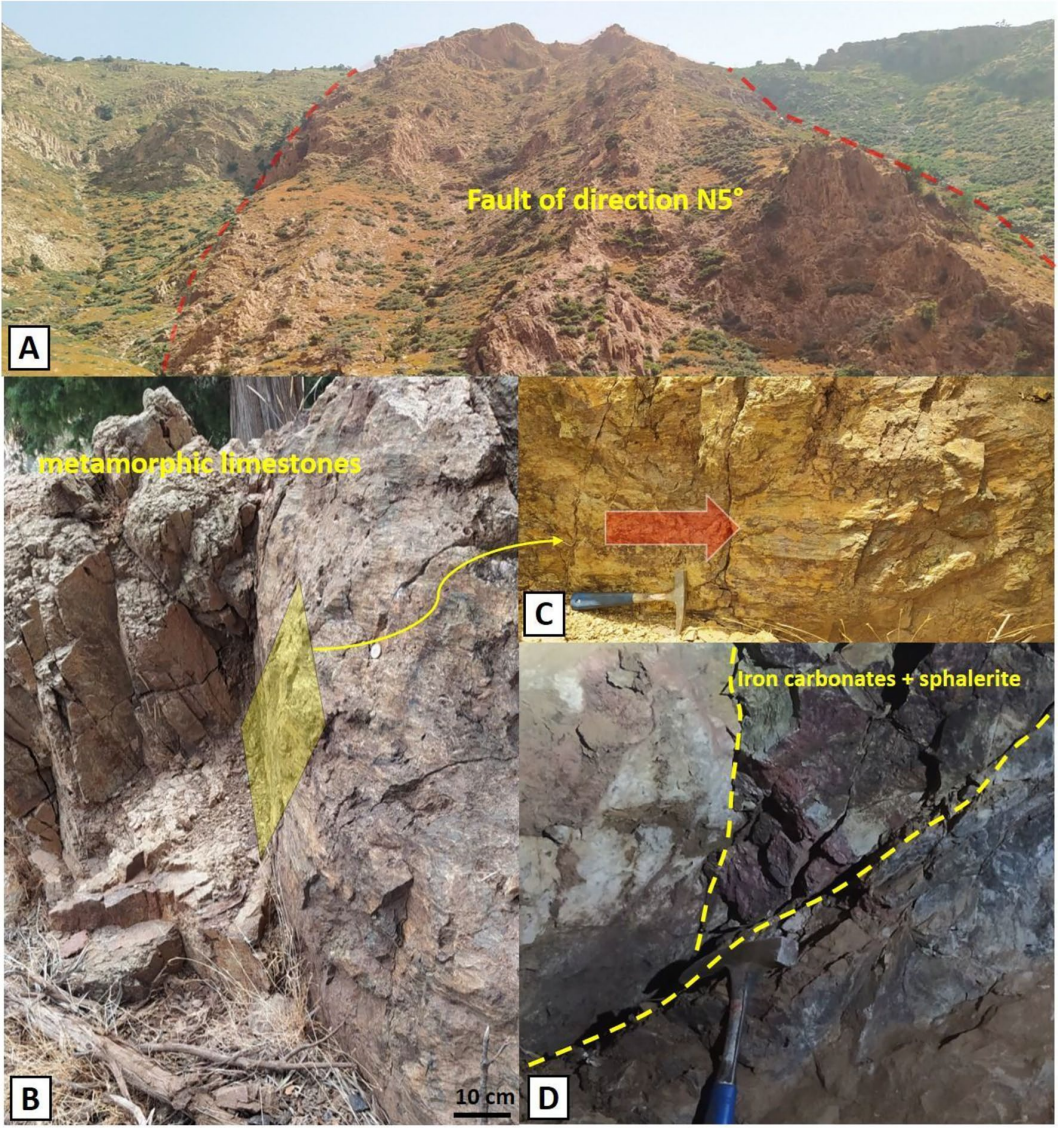
Structural control of Adebdi Zn-Pb mineralization. (**A**) N5° regional fault. (**B**,**C**) Sinister play fault controlling mineralization. (**D**) Columnar Zn-Pb mineralization.

In magnetic Area II, several mining indices containing Copper, Lead, Zinc, Molybdenum, and Barium are found near or along the boundary of Cambrian geological formations, in contact with the Tichka granite massif. These formations, composed of metagraywackes, siltshales, and limestones, exhibit relatively weak magnetic characteristics. From a mining perspective, these formations constitute the host rocks for mineralizations formed by hypothermal and pyrometasomatic processes, intimately related to regional tectonic evolution during the Hercynian orogeny and emplacement of the Tichka granite. A notable example of such mineralizations is found in the Ikissane and Adebdi deposits, where vein mineralizations traverse Lower Cambrian metamorphic limestones (Fig. 14D). The structural control of these mineralizations is mainly associated with the magnetic lineament MML4 and its satellite lineaments (Fig. 7), with a general NNE-SSW direction (Fig. 14B,C). Towards the east, a group of copper and barium mineralizations is associated with faults having NE-SW and E-W orientations (Fig. 14A). Among the major lineaments influencing these mineralizations, notable are MML1 (recognizable on the surface by the Tizi n'Test fault zone) and MML2 (related to the Arg fault zone) (Fig. 10). Besides base metals, numerous occurrences of albitite are exploited along the Tizi n'Test fault zone. The albitites, described by^2^, occur in Cambrian formations and are associated with the late hydrothermal episode of Cambrian volcanism, unrelated to the formation of the Tichka granite. These albitized formations are found in the Taghouacht, Jbel Boufounas, and Haute Goudacha areas.

**Figure 15:**
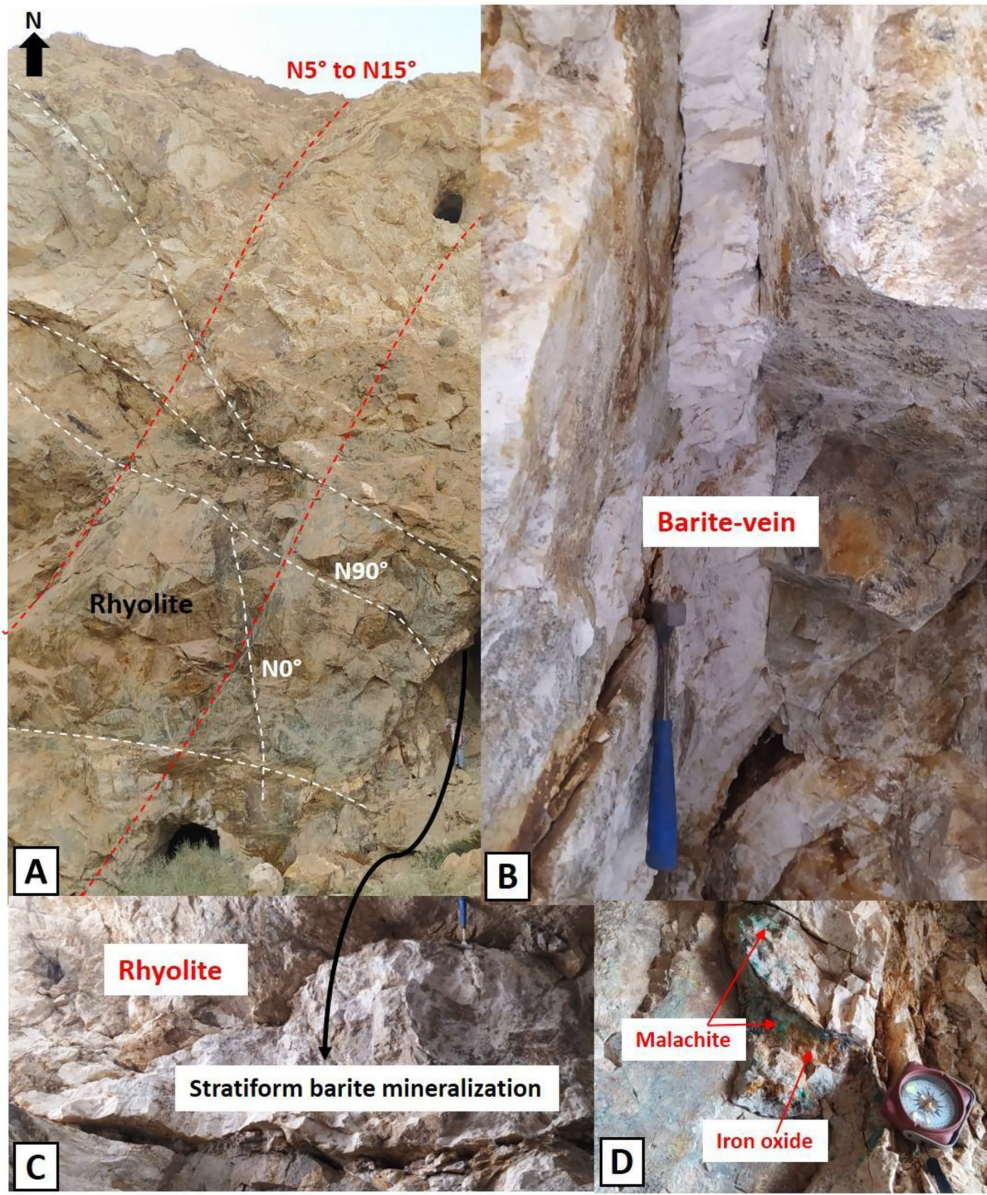
Morphology of barite mineralization in the Taghouacht deposit. (**A**) Two brittle structures mapped in Lower Cambrian rhyolites controlling mineralization. (**B**) N5° vein mineralization. (**C**) E-W cluster mineralization. (**D**) Copper carbonates associated with vein mineralization.

Finally, magnetic Area III is characterized by the presence of positive magnetic anomalies. These anomalies are associated with basic rocks in the southern part of the Tichka massif (Fig. 15), containing magnetic minerals such as ferromagnesian, as well as with Cambrian geological formations characterized by facies rich in volcanic and volcaniclastic materials. The elongated nature of these anomalies suggests they are likely related to magnetic materials along major NE-SW oriented faults, at depths below 400 m (Fig. 8). This orientation intersects with another direction clearly highlighted in Fig. 7, located in the Ifri n'Janjar mining district. In this zone, copper mineralization is associated with veins oriented E-W to NE-SW (Fig. 15A). These veins are controlled by ductile shear zone^1^, oriented NNW, and result from the final phase of the Hercynian orogeny. Additionally, southwest of the Tichka pluton, two directions of magnetic lineaments (Fig. 10A) could facilitate the flow of hydrothermal fluids. These fluids are likely related to the origin of copper and molybdenum mineralizations present in Cambrian metamorphic rocks, similar to those observed east of this massif. Lastly, south of the massif, the barium deposit at Taghouacht (Fig. 15B,C) is located in an area with negative magnetic texture, associated with NE-SW oriented magnetic lineaments which constitute satellites of the Tizi n'Test fault zone.

#### Validation

Due to ambiguity in the potential field interpretation, geological and structural mapping based solely on magnetic field data analysis is not sufficient for both geoscientific and economic applications, but other geological and geophysical results constrain the proposed interpretations. This approach provides a detailed view of the spatial distribution of geological formations and subsurface structures. It is essential for detecting high-potential mineralization zones, especially those associated with hydrothermalism and tectonics^76,79^. However, the significance of these studies is not limited to data analysis; it also requires on-ground validation to corroborate the results obtained from potential field data. In this regard, geological verifications were conducted on the ground at several promising sites distributed within three distinct magnetic domains.

The first study site is located southeast of the village of Tata n Yacoub, in magnetic domain I. This zone was chosen due to detected anomaly contrasts and magnetic lineaments, suggesting the possible presence of significant geological structures and mineralizations. On-site, significant tectonic structures primarily oriented along an N70°(Fig. 13A) axis were identified. These structures are associated with minor faults containing copper mineralizations and barite veins within Cambrian limestone formations (Fig. 13B–D).

The second site is located in the Albdi region, within magnetic domain II. This zone also exhibited signs of significant deformation corridors during potential field data analysis (Figs. 7 and 10A). On-ground investigations and analysis of previous mining works revealed that mineralization occurs within a structural complex marked by a sinistral fault and is bounded to the south by an N5°(Fig. 14A) oriented fault with sub-vertical dip. This zone corresponds to a minor lineament derived from a major lineament, the MML4. In this site, mineralized veins with NE-SW orientation traverse Cambrian metamorphic limestones along their stratification. They mainly consist of iron-bearing carbonates and host a lead–zinc mineralization dominated by sphalerite(Fig. 14D).

The third study site is located in the Tghoucht deposit, within magnetic domain III. Geological verifications on the ground focused on characterizing barite mineralizations and identifying associated geological structures. The collected data indicate that barite mineralization (Fig. 15B,C) is related to two main structural orientations: (i) an E-W orientation with a southward dip of 50°, influencing mineralization localization(Fig. 15A). The layers are stratified and rich in calcite, exhibiting a brecciated texture composed of rhyolite fragments and calcite crystals associated with copper sulfides such as bornite and chalcopyrite (Fig. 15D). (ii) another N5° orientation with a westward dip of 80°, guiding vein-type mineralization formation. These fractures sometimes deform mineralization into masses and are associated with copper carbonate.

## Conclusions

In conclusion, geological and structural mapping based on magnetic field data analysis proves to be a valuable tool for identifying and characterizing high-potential mineralization zones. In this study, analysis and interpretation of aeromagnetic data in the Tizi n'Test region allowed us to divide the study area into three magnetic domains, each correlated with unique geological characteristics: (i) long-wavelength anomalies associated with Ediacaran basement formations, (ii) generally negative anomalies interpreted as magnetic signatures of Phanerozoic sedimentary cover formations, and (iii) short-wavelength anomalies associated with basic magmatic formations of the Tichka massif. Furthermore, magnetic filtering results revealed that the studied area is characterized by predominant lineament directions, notably NE-SW, ENE-WSW, and E-W with depths ranging from 50 to 3500 m. These lineament orientations showed a strong correlation with the distribution of observed mineral occurrences on the surface. This correlation between lineament directions and mineral occurrence distribution reinforces the hypothesis that tectonic structuring of the area played a determinant role in mineral concentration and localization. The structural pattern elaborated and categorized by fractal modelling C-A highlighted several key areas conducive to mineralization. These areas are favoured by the channelling effects of hydrothermal fluid lineaments, among which we can mention: (i) the magnetic contrast zone located between the AMHA and OB blocks, materialized by the Ouchden fault; (ii) the magnetic contrast zone between the Tichka granite and surrounding lithologies; (iii) the Tizi n'Test fault zone; and (iv) the magnetic axes associated with hydrothermal alterations in the northwest of the study area. The study underscores the potential of using aeromagnetic data in conjunction with ground geology to discover mineral exploration targets, emphasizing the critical role of structural controls in mineral deposit processes.

The findings presented here have significant implications for mineral exploration strategies in the Tizi n'Test region and similar geological settings.

## Data Availability

The data that support the findings of this study are available from the corresponding author upon reasonable request.
